# 

**Published:** 1893-04

**Authors:** 


					﻿Vol XIII.
APRIL, 1893.
NO. 4.
THE
HOMEOPATHIC pHY^lClApJ,
A Monthly Journal of Homoeopathic Materia Medica
and Clinical Medicine.
“ He is a freeman whom truth makes free, and all are slaves beside."
“ Seek the Truth: come whence it may, cost what it will."
EDITED AND PUBLISHED BY
WALTER M. JAMES, M. D.
PHILADELPHIA :
No. lies SPRUCE STREET.
jtOHrDON:
ALFRED HEATH & CO., Sole Agents for Great Britain,
114 Ebury Street, Eaton Square.
Yearly Subscription, $2.50. invariably in advance. 8ingle numbers, 25 cts.
Entered at the Philadelphia Peet-Offiee aa Second-Claaa Mail Matter.
				

